# Magnetic Field Measurements during Magnetic Pulse Welding Using CMR-B-Scalar Sensors

**DOI:** 10.3390/s20205925

**Published:** 2020-10-20

**Authors:** Voitech Stankevic, Joern Lueg-Althoff, Marlon Hahn, A. Erman Tekkaya, Nerija Zurauskiene, Justas Dilys, Jonas Klimantavicius, Skirmantas Kersulis, Ceslovas Simkevicius, Saulius Balevicius

**Affiliations:** 1 Center for Physical Sciences and Technology, Department of Functional Materials and Electronics, Sauletekio ave. 3, LT-10257 Vilnius, Lithuania; nerija.zurauskiene@ftmc.lt (N.Z.); justas.dilys@ftmc.lt (J.D.); jonas.klimantavicius@ftmc.lt (J.K.); skirmantas.kersulis@ftmc.lt (S.K.); ceslovas.simkevicius@ftmc.lt (C.S.); saulius.balevicius@ftmc.lt (S.B.); 2 Faculty of Electronics, Vilnius Gediminas Technical University, Naugarduko 41, LT-03227 Vilnius, Lithuania; 3 Institute of Forming Technology and Lightweight Components, TU Dortmund University, Baroper Str. 303, D-44227 Dortmund, Germany; joern.lueg-althoff@iul.tu-dortmund.de (J.L.-A.); marlon.hahn@iul.tu-dortmund.de (M.H.); erman.tekkaya@iul.tu-dortmund.de (A.E.T.)

**Keywords:** magnetic pulse welding (MPW), magnetic field sensor, magnetic field dynamics, welding process monitoring

## Abstract

The possibility of applying CMR-B-scalar sensors made from thin manganite films exhibiting the colossal magnetoresistance effect as a fast-nondestructive method for the evaluation of the quality of the magnetic pulse welding (MPW) process is investigated in this paper. This method based on magnetic field magnitude measurements in the vicinity of the tools and joining parts was tested during the electromagnetic compression and MPW of an aluminum flyer tube with a steel parent. The testing setup used for the investigation allowed the simultaneous measurement of the flyer displacement, its velocity, and the magnitude of the magnetic field close to the flyer. The experimental results and simulations showed that, during the welding of the aluminum tube with the steel parent, the maximum magnetic field in the gap between the field shaper and the flyer is achieved much earlier than the maximum of the current pulse of the coil and that the first half-wave pulse of the magnetic field has two peaks. It was also found that the time instant of the minimum between these peaks depends on the charging energy of the capacitors and is associated with the collision of the flyer with the parent. Together with the first peak maximum and its time-position, this characteristic could be an indication of the welding quality. These results were confirmed by simultaneous measurements of the flyer displacement and velocity, as well as a numerical simulation of the magnetic field dynamics. The relationship between the peculiarities of the magnetic field pulse and the quality of the welding process is discussed. It was demonstrated that the proposed method of magnetic field measurement during magnetic pulse welding in combination with subsequent peel testing could be used as a nondestructive method for the monitoring of the quality of the welding process.

## 1. Introduction

Magnetic pulse welding (MPW) is a collision welding process, which uses a high velocity impact to join the two metals. This method allows for the joining of similar and dissimilar metals without the input of external heat and without any critical formation of their intermetallic phases [1]. Sheets, profiles, and tubes can be processed. During the MPW process in tubular configuration, the parts being welded (the flyer tube and the fixed inner parent) are positioned inside a tool coil. During the fast discharge of the capacitor banks via the coil, a magnetic field is generated around the coil, which leads to eddy currents in the electrically conductive flyer part positioned in close vicinity to the coil. To concentrate the magnetic field, e.g., in a smaller joining zone, field shapers are used, which are positioned between the coil and the flyer tube. The eddy currents induced in the flyer create an opposite magnetic field and a repulsive Lorentz force. This force causes the flyer to quickly accelerate plastically and to impact with the inner parent at an extremely high velocity resulting in a metallic bond. It should be noted that in the gap between the shaper and flyer, the two fields have the same direction and the total field is the sum of the field generated by the field shaper and the eddy current. To ensure a stable impact welding process and to optimize the used energy, the impact conditions need to be carefully controlled (see Pereira et al. [2]).

The parameters used for the definition of the welding quality are often displayed in welding windows, see Crossland et al. [3]. In the case of MPW, these depend on the geometry of the joining setup and on the materials being welded. These parameters, however, are dynamically changing during the welding process due to the transient magnetic pressure. It was demonstrated that these parameters must be adjusted, depending on the properties of the applied materials. For example, Psyk et al. [4] presented the results of joining copper alloys with aluminum alloys. Shanthala et al. [5] optimized the MPW process for joining two steel parts. The magnetic pressure and discharge pulse frequency are the key parameters responsible for driving the flyer towards the parent part (see Lueg-Althoff et al. [6] and Psyk et al. [7]). Since the magnetic pressure changes over time, the correlation between the impact conditions and the welding properties requires suitable analysis methods (see, for example, the overview of Bellmann et al. [8]).

One of the simplest ways of obtaining information about the electromagnetic processes which take place during MPW is to measure the waveform of the current in the magnetic coil. The current waveform is a result of the capacitor discharge through the resistive and inductive elements of the welding system (the coil, field shaper, workpiece, transmission line, etc.). In the case of a coil with a non-deforming workpiece, the current oscillates according to a damping law. However, due to the change of inductance of the deforming flyer and due to the changes of the mutual inductance between the coil and the flyer during its movement, the waveforms of the current through the coil strongly differ. The change of this waveform could be used for the evaluation of the MPW quality. However, this method is not sensitive enough when the welding is realized in a narrow part of the workpiece where a field shaper is used. For this reason, it has been suggested that the magnetic field could be measured directly in the small gap between the field shaper and the workpiece. For this purpose, various research groups have developed custom probes such as small fiber-optic sensors based on the magneto-optic Faraday effect and the Hall sensor (Bellmann et al. [8]) for the measurement of the strength of the magnetic field between sheets during MPW (see Merte et al. [9]), and the induction coils for the measurement of the magnetic field in the active zone of the coil (Broeckhove et al. [10]). Unfortunately, the use of such sensors does not completely solve the challenges that prevent detailed studies of the impact and welding procedure in MPW. These include the limited accessibility of the joining zone due to its overlap configuration, interference of the magnetic fields with the electrical output signals, and the extremely short process duration, which is only a few microseconds.

An alternative to these measurement methods could be an analytical investigation of the magnetic field dynamics in the gap between the coil or field shaper and the flyer during the MPW process. There have been several analytical calculations, which were performed for this purpose, as summarized by Psyk et al. [7]. For example, Nassiri et al. [11] showed that, by using an analytical model for a single turn coil, one can predict the magnetic pressure on the flyer as well as its velocity during forming. In this case, the Lorentz force $\vec{F}$, which acts on the flyer, can be calculated as the vector product of the magnetic flux density $\vec{B}$ of the coil (field shaper) and the eddy current induced in the flyer with a current density $\vec{J}$

(1) $$\vec{F}=\vec{J}\times \vec{B}$$

The current density $\vec{J}$ is related to the magnetic field $\vec{H}$ through a partial derivative (from Maxwell’s equations) in radial direction *r* (from field shaper to tubular flyer)

(2) $$\vec{J}=-\frac{\partial \vec{H}}{\partial r}$$

Therefrom, the volume force, which acts on the non-magnetic flyer, can be calculated as

(3) $$\vec{F}=-\frac{\partial \vec{H}}{\partial r}\;\vec{B}=-\frac{\partial \vec{H}}{\partial r}\;\mu_{m}\vec{H}=-\frac{1}{2}\;\mu_{m}\;\frac{\partial \left({\vec{H}}^{2}\right)}{\partial r}$$

where $\mu_{m}$ is the permeability of the material. The effective pressure acting on the flyer surface is determined as the integral of the body force over the thickness *d* of the flyer. Moreover, taking into account that due to the skin effect the penetrated magnetic field into the opposite side of flyer is neglected, the magnetic pressure can be simplified to (see Psyk et al. [7] and Nassiri et al. [11])

(4) $$P=\int_{0}^{d}\vec{F}\partial r=\frac{1}{2}\;\mu_{m}H_{gap}^{2}$$

where *H_gap_* is magnetic field strength in the gap.

However, this analytical method requires empirical values, which must be obtained experimentally. For this reason, the measurement of the magnetic field dynamics during electromagnetic processing of the workpieces becomes of great importance. However, it is not easy to perform such measurements due to the specific requirements for the magnetic sensors as discussed above.

During the last few decades, it has been demonstrated that magnetic field sensors based on nanostructured lanthanum manganite films exhibiting the colossal magnetoresistance (CMR) phenomenon could be used for the measurement of high pulsed magnetic fields in very small volumes (10^−^^2^ mm^3^), see Žurauskienė et al. [12]. These sensors can measure the magnetic field magnitude independent of the magnetic field direction (CMR-B-scalar sensors), see Stankevič et al. [13]. These were used for the measurement of magnetic field distribution, see Liebfried et al. [14], and the diffusion processes in railguns, see Schneider et al. [15]. Moreover, Haran et al. [16] demonstrated that during the diagnostics of a high-velocity railgun projectile launch, these sensors can provide a measure of the total magnetic field present at a particular location in the launcher and are a suitable instrument for measuring this field with high resolution in both space and time. In addition, these CMR-B-scalar sensors were used for the measurement of highly inhomogeneous transient magnetic fields during coilgun experiments [17] and the distribution of these magnetic fields inside nondestructive pulsed magnets [18]. Due to the polycrystalline structure of such manganite films, the responses of these sensors do not saturate when subjected to high fields and they were capable of measuring magnetic fields up to the megagauss range (91.4 T), see Balevicius et al. [19]. Thus, the application of CMR-B-scalar sensors for the measurement of magnetic fields during magnetic pulse welding is very promising.

In this work, it was investigated how fast, small size CMR-B-scalar sensors could be used for the measurements of the magnetic field magnitudes in the gap between the flyer and the field shaper during MPW and how they can provide quick, non-destructive evaluations of the weld quality.

## 2. Experimental Setup

**Figure 1:**
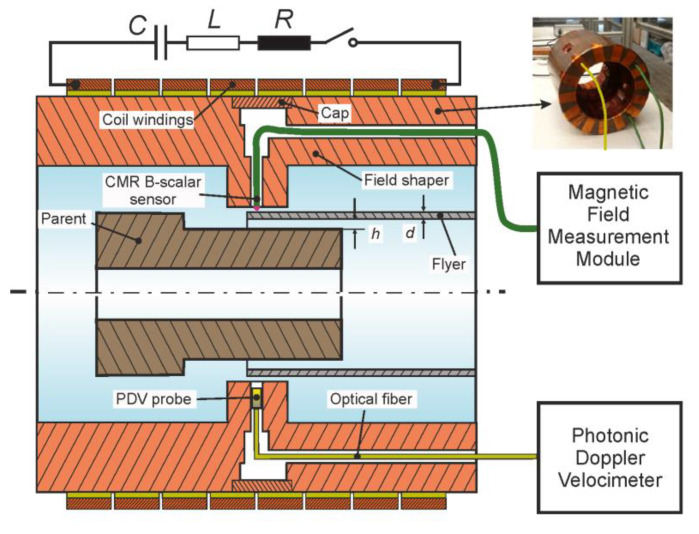
Setup for magnetic pulse welding of tubes to cylinders with a CMR-B-scalar probe for the measurement of the magnetic field and a PVD sensor. In the upper right corner, a picture of the field shaper with the integrated two CMRs and one PDV sensor.

A schematic diagram of the setup used for the MPW process investigation is shown in Figure 1. This setup was installed at the Institute of Forming Technology and Lightweight Components of TU Dortmund University. It consisted of a compression coil, a field shaper, and the workpieces (parent and flyer tubes). A Poynting SMU 0612 FS pulse power generator was used for the generation of the electrical current through the tool coil as was shown in more detail in Lueg-Althoff et al. [20]. This current was recorded using a Rogowski coil type CWR 3000 B of Power Electronic Measurements Ltd. in combination with a LeCroy Waverunner 104 MXi digital oscilloscope. The discharge energy *E* was set to values between 2.0 and 5.0 kJ. The field shaper (see Figure 1) had an outer axial length of 110 mm and a 15 mm length concentration zone, which was used to adapt the solenoid compression coil to the outer diameter of the tubular specimens. The compression coil type Poynting SMU-K97-8/90 contained eight windings with a 48.5 mm inner radius and an axial length of 91.6 mm. The welding experiments were performed at different discharge energies of the capacitor (2, 3, 4, and 5 kJ), varying the wall thickness of the flyer (1, 2, 2.5 mm) and the standoff distance between flyer and parent (1.5 and 2 mm). The cylindrical parent made of steel C45 was positioned inside the flyer tube during these experiments. For comparison, additional experiments were performed with a rigid rod and a freely compressing tube.

The weld quality was examined using the standard peel testing method for the determination of the joint strength of the MPW specimens [8]. After welding, small kerfs were cut into the aluminum flyer and the resulting strips were peeled at an angle of 90 degrees off the parent. A separation of the whole strip indicated a poor weld seam, while a failure in the weaker base material indicated a sound weld. It should be noted that after the experiments, a manual peel test was performed in the workshop, because a qualitative result was sufficient at this stage of the study.

The dynamics of the magnetic field at the gap between the field shaper and the flyer during electromagnetic compression and MPW were measured using a high-pulsed magnetic field measurement system based on CMR-B-scalar sensors, which were developed at the Center for Physical Sciences and Technology, Vilnius, Lithuania. The system consisted of a CMR-B-scalar probe and an electronic measurement module. The probe was designed as a flexible, 3 mm in diameter and 1 m long screened cable with a 1 × 0.5 × 0.25 mm^3^ volume CMR-B-scalar sensor at its tip. The sensor was made from manganite film, which exhibits a negative colossal magnetoresistance effect. Due to Mn excess, it has an increased temperature range of operation from 0 °C to 90 °C (see Žurauskienė et al. [21]). The active material in the sensor was a 0.4 μm thick nanostructured La_0.82_Sr_0.18_Mn_1.15_O_3_ film grown on a polycrystalline Al_2_O_3_ substrate by pulsed injection metal-organic chemical vapor deposition (PI MOCVD), see Zurauskienė et al. [22]. The active part of the sensor’s material was 400 μm in width and 50 μm in length. Thus, the effective measurement volume was ~10^−2^ mm^3^.

The CMR-B-scalar measurement module used for data acquisition and recording was an electronic device specially protected against harsh electromagnetic conditions by an inner 4 mm thick steel box and an external 1.5 mm thick aluminum box. The analog signal of the sensor in this module was digitized using a 16-bit analog-digital converter with a sampling rate of 25 MS/s. This high-pulsed magnetic field measurement system, consisting of the CMR-B-scalar sensor and the electronic module, was able to measure the magnetic field in frequency ranges from DC to 100 kHz with a resolution of the magnetic field value of not less than 10 mT. This was sufficient to achieve good time resolution during the measurement of the magnetic pulses with durations of several hundred microseconds. The measured signal was processed and recorded in the memory of the module. Then, the recorded signal was transmitted to a personal computer by means of a fiber optic link.

During the experiments, the magnetic field probe was integrated into the field shaper by means of several boreholes and pockets (see Figure 1). Thus, the magnetic field between the field shaper and the workpiece could be measured in the center of the concentration area at a position 1 mm radially deep inside the field shaper.

The radial velocity, as well as the displacement of the specimens was measured by means of a photonic doppler velocimeter (PDV) as described by Bellmann et al. [8]. This laser-based measurement technology was used because it is capable of measuring high velocities without being disturbed by the acting magnetic fields. A small size collimator probe was integrated into the field shaper (see Figure 1) as explained in Lueg-Althoff et al. [20].

## 3. Results and Discussion

### 3.1. Measurement of Magnetic Field Dynamics during MPW

**Figure 2:**
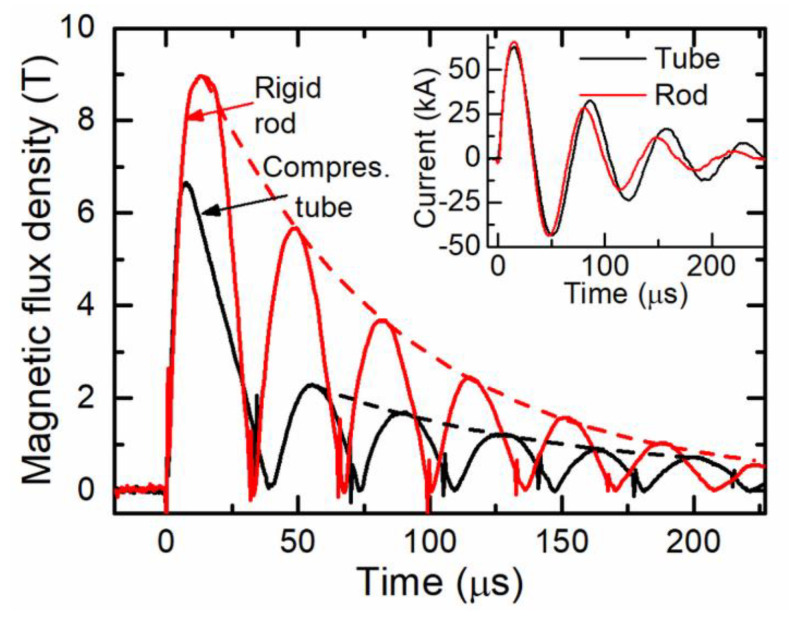
Magnetic field dynamics between the field shaper and the flyer part. The red curve marks the rigid rod; the black curve marks the 1.5 mm thick Al tube. Inset: the discharge current during tube compression is shown by the black line; during experiments with a rigid rod, by the red line.

Prior to the study of the MPW process, the magnetic field dynamics in the gap between the field shaper and a rigid aluminum cylinder as well as during the free compression of an aluminum tube were investigated. The results of these investigations at a charging energy of 4 kJ are shown in Figure 2 for a rod (aluminum EN AW-6060, delivered by BIKAR-Metalle, Bad Berleburg, Germany) with a diameter of 40 mm and an aluminum tube with the same outer diameter and a wall thickness of 1.5 mm. The coil current for both cases is shown in the inset of Figure 2. The periodically changing magnetic field waveform is unipolar, which is a result of the CMR-B-scalar sensor measurement, because it measures only the absolute value of the magnetic flux density *B*. In the case of the aluminum rod, the amplitudes of all the magnetic field oscillations change according to a damping law (dashed line) with the exponential time constant *τ* = 75.5 µs. It should be noted that the amplitudes of the currents also change according to this law. However, in the case of tube compression, after the first half-wave pulse, the amplitude of the next half-wave significantly decreases, and the magnetic field oscillates according to a damping law with the exponential time constant *τ* = 90.7 µs.

These differences are due to the tube deformation during the first half-wave of the current, which causes a significant increase of the inductance of the system consisting of the tube and the field shaper. This is also confirmed by the decrease of the frequency of the magnetic field oscillations (see Figure 2). According to Kleiner et al. [23], during the compression process, when the flyer deformation starts, the total inductance of the consumer load (coil-tube) in the discharge circuit increases, which leads to a decrease of the oscillating current frequency and the amplitude. However, due to the presence of the field shaper, which is used in addition to the tool coil, the deformation of the tube only marginally affects the total inductance of the system (see Henselek et al. [24]). Consequently, the current in the coil changes only slightly (see inset in Figure 2). Nevertheless, this leads to a significant decrease of the magnetic field amplitude in the gap between the field shaper and the compressed tube. Moreover, as can be seen in Figure 2, during the compression of the tube, the temporal course of the first half-wave of the magnetic field is different from that of the rigid rod. Here, the maximum of the magnetic field is achieved much earlier than the maximum of the coil current. This means that despite the increase in coil current, the magnetic field in the gap decreases due to the deformation of the flyer.

**Figure 3:**
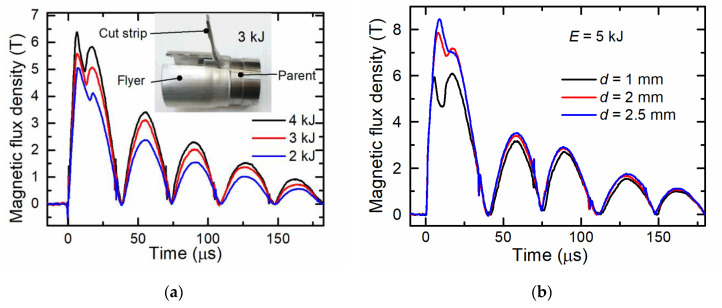
Magnetic fields between the field shaper and the aluminum flyer tube at different charging energies when tube wall thickness is *d* = 1.5 mm, the joining gap is *h* = 1.5 mm (**a**) and the charging energy is 5 kJ, also when the wall thicknesses are *d* = 1, 2, 2.5 mm (**b**).

To study the influence of the charging energy *E* on the magnetic field oscillation waveforms during MPW of an aluminum flyer with a steel parent, experiments were performed at different charging energies (see Figure 3a) and with flyers having different wall thicknesses (see Figure 3b). The results of these investigations with a flyer wall thickness of 1.5 mm at charging energies of 2, 3, and 4 kJ are shown in Figure 3a. The first half-wave in all these welding experiments had two peaks. The first peak was similar to that observed during the free tube compression (see Figure 2). The amplitude of these peaks depended on the capacitor charging energy, namely, the lower the charging energy, the lower was the amplitude of the magnetic field peak, and the maximum of these peaks was reached later. As mentioned before, the magnetic inductance at the measuring point is the interference of the field generated by the field shaper and the induced current in the flyer. The second component, among other factors, depends on the distance between the field shaper and the flyer. The greater this distance, the smaller becomes the contribution of the magnetic field of the flyer. At a certain velocity of the flyer, the increase in time of the magnetic field in the field shaper (with the coil current still increasing) was compensated by a decrease of the magnetic field induced in the flyer. The first peak was obtained in this way. After the first peak was reached, the magnetic flux density dropped to some local minimum value, then increased up to the second peak and finally dropped to zero. Moreover, the minimum between these two peaks in time was achieved later and the relative value of the second peak was lower when a lower charging energy was used. After the end of the first half-wave, the following pulses continued to oscillate according to the damping law.

**Figure 4:**
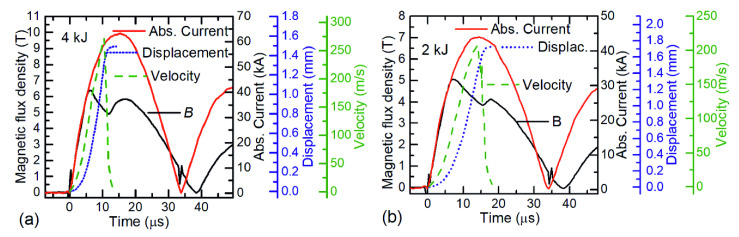
Magnetic field dynamic between the field shaper and Al tube is marked by the black line, the current of the coil by the red line, the displacement of the walls of tube by the dotted blue line and the velocity of the tube wall by the dashed green line. Tube walls thickness was 1.5 mm, the capacitor charging energy was 4 kJ (**a**) and 2 kJ (**b**). The parent was made of steel.

To understand the dynamics of the magnetic flux density during the welding process, the processes that take place during the first half-wave of the discharge current were considered. Figure 4 shows the current in the coil and the changes of the magnetic field magnitude in the gap between field shaper and flyer.

The displacement of the flyer as well as its velocity at 4 kJ and 2 kJ charging energies are presented in Figure 4a,b, correspondingly. A comparison of the flyer displacement over time and the magnetic flux density *B* waveform shows that the local minimum of *B* is reached at the collision of the flyer with the parent. At the instant of impact, the velocity of the flyer achieves its maximum value and then drops sharply. For *E* = 4 kJ (Figure 4a), the initial collision time is 11 µs after the start of the current pulse and the maximum impact velocity of the flyer is *v*_i_ = 272 m/s. The total displacement of the flyer is 1.5 mm (i.e., the initial gap) and the deformation process is finished after 14 µs. The minimum magnetic flux density is achieved when the flyer velocity decreases to *v*_i_ = 120 m/s. As it was noted above, the appearance of the first peak and the subsequent decrease of the magnetic field was the result of the flyer deformation caused by the magnetic pressure. In this case, an increase of the system’s inductance caused by the progressing deformation of the flyer slowed down the magnetic field so strongly that an increase of the current in the coil was not able to compensate for this decrease. However, when the velocity was decreased, the influence of the coil current on the magnetic field dominated and an overall increase of *B* was observed. For this reason, at the time instant of magnetic field minimum, the current did not yet reach its maximum value and continued to grow, which caused the appearance of the second peak in the field dynamics.

It was found that there is a relationship between the charging energy *E*, the time instant (*τ*_m_) of the magnetic field minimum in the double peak waveform and the welding quality. When *E* was 4 kJ, 3 kJ, and 2 kJ, the *τ*_m_ was 11 µs, 12.5 µs, and 15 µs, respectively. The manual peel test showed that at *τ*_m_ ≤ 12.5 µs, the cut strip of the flyer material could not be separated from the parent, which indicated a good weld quality. A picture of the tested specimen after MPW at *E* = 3 kJ; *τ*_m_ = 12.5 µs is shown in the inset of Figure 3. Meanwhile, the same analysis performed at *E* = 2 kJ; *τ*_m_= 15 µs (see Figure 4b) showed an insufficient weld strength, because the flyer strip was easily peeled off from the parent.

The influence of the flyer wall thickness on the magnetic field dynamics was studied by charging the capacitor up to 5 kJ and using flyers with wall thicknesses of 1, 2, and 2.5 mm. The results are shown in Figure 3b. It can be seen that a flyer with a wall thickness of 1 mm impacts with the parent after *τ*_m_ = 11 μs (minimum of the curve), while the flyer with a wall thickness of 2.5 mm impacts with the parent after *τ*_m_ = 16 μs when the coil current had already reached its maximum value and started to drop. Moreover, in the case of flyer with a thickness of 2.5 mm, the energy was not high enough for sound welding of the parts.

Therefore, by analyzing the waveform of the magnetic field over time, one can qualitatively determine the quality of the MPW process taking into account the following parameters: the time instant and amplitude of the first peak and the time instant of the minimum. As stated before, the flyer deformation begins when a critical magnetic pressure is reached. The first maximum is a result of the compensation of the increasing magnetic field in the field shaper effected by the decreasing magnetic field induced in the flyer. The maximum magnetic pressure can be evaluated from this maximum value. However, it is difficult to determine a clear relationship between the time instant of the first maximum and its value with the weld quality. The reason is that the position and value of this maximum depends on the increasing coil current, the velocity of the flyer, its mechanical properties, etc. For example, for flyers with a fixed wall thickness, it was obtained that at a higher charging energy, an earlier and higher maximum is achieved and the welding quality is increased (see Figure 3a). However, the time intervals between these peaks differ only a little. For flyers with different wall thicknesses (see Figure 3b), the first maximum of 6 T was achieved after 6 μs at a charging energy of 5 kJ when a flyer with a wall thickness of 1 mm was used. However, when a flyer with a wall thickness of 2.5 mm was used at the same energy, this was achieved after 11 μs despite of the higher value of the first peak (about 8.3 T). Moreover, the time instant of the magnetic field minimum shows the time of the flyer impact with the parent. Based on the obtained data, the flyer velocity at the moment of its collision with the parent can be determined. After the beginning of the deformation, the velocity of the flyer changes almost linearly. According to Lueg-Althoff et al. [6], the time of impact of flyer with parent is a particularly important parameter in the MPW process. Thus, knowing the joining gap and the time until impact, one can estimate the velocity of the flyer. The later the local minimum is reached, the lower is the flyer velocity shortly before the impact with the parent. The acting pressure on the flyer part can be assessed by analyzing the velocity change during impact. This has been done by Lueg-Althoff et al. [6] by evaluating the flyer velocity change upon impact via the PDV curves. However, by introducing the CMR-B scalar measurement system as a novel and fast analysis method of MPW processes, one can get information not only about the flyer movement, but also about the magnetic field values and dynamics. Therefore, the combination of a few parameters—such as the first peak maximum, its time-position, and the time instant of the waveform minimum—could be used as an indication or ‘fingerprint’ of the welding quality for known MPW setups with predetermined tool parameters, such as the gap between the flyer and the field shaper, the standoff between the flyer and the parent and the flyer wall thickness.

The obtained results show that the analysis of the dynamics of the magnetic field in the vicinity of the field shaper gives necessary information about the processes taking place during magnetic welding, which cannot be obtained by direct measurement of the coil current, which was suggested by Henselek et al. [24], Zhang et al. [25], and Beerwald et al. [26]. In addition, by analyzing the data obtained by measuring the dynamics of the magnetic field and comparing this data with the results of a physical check of the weld quality, it will be possible to optimize the charging energy of the capacitors for each case. For example, it can be seen that for welding a flyer with a wall thickness of 1 mm it is not necessary to charge the capacitor to 5 kJ, since it impacts the parent when the current in the coil has not yet reached its maximum. In addition, the proposed magnetic field measurement system can be not only used for analyzing the processes occurring during MPW, but also for the monitoring of critical points of magnetic field dynamics (such as the magnitude and time-position of the first maximum and minimum) determined in advance for selected MPW system. The CMR-B-scalar sensors are small in size and not sensitive to the direction of the magnetic field, which greatly facilitates their installation. They are also fast and can measure short pulses of the magnetic field. In addition, in comparison with PDV sensors, they do not require special signal processing, as the entire dynamic of the magnetic field is immediately displayed on the monitor screen.

### 3.2. Numerical Simulations of Magnetic Flux Density in the Gap between Field Shaper and Workpiece

**Figure 5:**
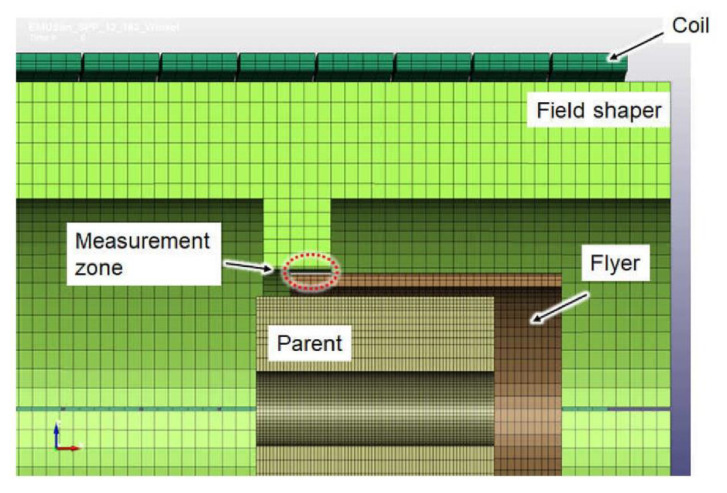
Setup in LS-DYNA simulation.

For numerical simulation of the magnetic field dynamics, 3D simulation models were created using the commercial software LS-DYNA (Lorenz et al. [27]). Although the experiments were carried out with flyers of different wall thicknesses, the simulation was performed for only one flyer geometry. Since the numerical simulation of high velocity forming processes like electromagnetic forming (or in this case MPW) is elaborate and challenging, the focus was put on a flyer with 2 mm wall thickness. For this geometry, the required high-strain rate material parameters had been accurately determined beforehand. Thus, a stable and informative numerical model could be evaluated. The simulation setup is shown in Figure 5.

In this setup, the flyer had *d* = 2 mm thickness, the initial gap between the flyer and the parent *h* was 1.5 mm, and the initial standoff between field shaper and flyer was 0.5 mm. Besides the mechanical material properties, an experimentally measured electrical current pulse through the coil was used as the input. The accuracy of numerical simulation models of high-velocity forming processes such as MPW strongly depend on the applied material models. The material data for the numerical simulations have been obtained by inverse characterization, like described in Lorenz et al. [27]. Since the applied field shaper features multiple axial and radial boreholes for the integration of the CMR-B-scalar probes and the PDV-sensors (see Figure 1), the creation of the mesh of the 3D simulation model was challenging. An exceptionally fine 3D-mesh was required, which drastically increased the simulation time. Therefore, the boreholes were not considered in the LS-DYNA simulations. Additionally, the influence of the boreholes and the position of the magnetic field sensor on the magnetic flux density values was evaluated by using COMSOL multiphysics simulations. The results of the COMSOL simulations are presented below.

**Figure 6:**
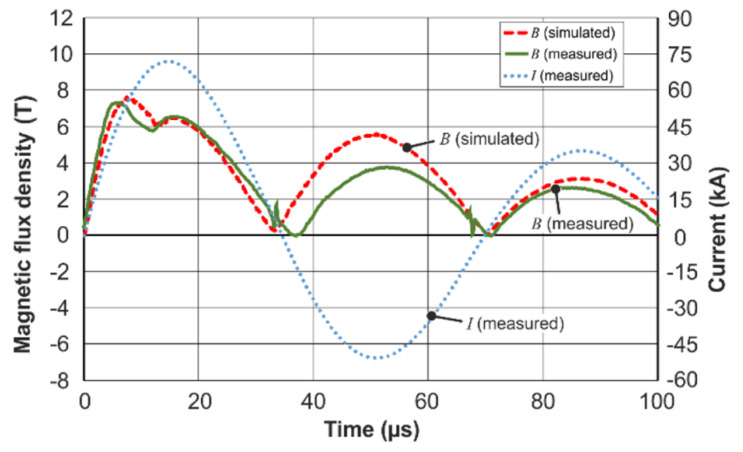
Magnetic field dynamics during the welding of an aluminum tube of wall thickness *d* = 2 mm with the steel parent. Scaled LS-DYNA simulation (scaling factor 0.3 is the red dashed line), the measurement is the green line and the current oscillations through the coil are the blue dotted line.

The comparison of the simulation results and the measurements of the magnetic field dynamics showed good qualitative coincidence between the simulation and the experiment (see Figure 6). Especially the temporal course during the first half-wave of the current pulse showed a promising accordance.

**Figure 7:**
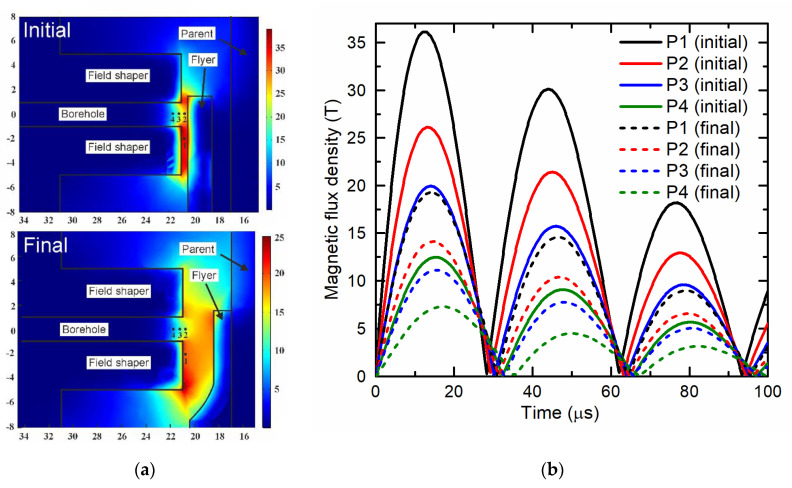
COMSOL simulation results of the magnetic field distribution at four positions in the gap between flyer and field shaper for the initial and final flyer deformation states ((**a**) strength of the magnetic field, (**b**) magnetic field oscillation). P1 is unaffected by the borehole, P2 is at the same radial position in front of the borehole, P3 and P4 is 0.5 and 1 mm inside the borehole, which is approximately the position of the probe in the experiments.

The previously discussed two peak behavior in the field intensity dynamics was found both in the measurements and the simulation. However, as mentioned before, the dynamic LS-DYNA simulations were performed without boreholes and the simulation results give significantly higher magnetic field values, than were obtained from the measurements using the CMR-B-scalar sensor. For example, the maximum flux density calculated for the time instant *t* = 7.1 µs (see Figure 6) was 25.05 T (not shown in the Figure), while the measured value was ≈ 7.48 T. To clear up the reasons for this discrepancy, time-harmonic 3D simulations were performed using COMSOL Multiphysics. The objective was an evaluation of the influence of the borehole and the position of the magnetic field sensor on the magnetic flux density. The geometries of the field shaper, flyer, and parent were considered in both their initial (not deformed) states and their final states after the collision of the flyer with the parent. The results presented in Figure 7 show that the borehole results in a significant local reduction of the magnetic field. Along the axis of the borehole, the magnetic field is inhomogeneous. A comparison of the maximum value of the magnetic field at Point 1 (at which the calculations by LS-DYNA were carried out) with the maximum at Point 4 (in which the sensor was placed), shows that these values differed by about three times. As it was mentioned before, the same three-time difference was obtained when comparing the simulation and measurement results during the welding process. Additionally, it can be seen that the increase of the standoff between the field shaper and the flyer due to electromagnetic forming leads to a decrease of the magnetic field intensity and a temporal shift of the maximum and minimum.

Therefore, the presence of a hole in the field shaper and the position of the sensor in this hole causes the discrepancy between the measured value and simulated magnetic field. Thus, it was decided that the LS-DYNA results need to be scaled with a factor of 25.05/7.48 to take into account the influence of the borehole (see Figure 7). Therefore, the data of the simulation results, presented in Figure 6, are scaled by this factor. This makes it possible to obtain not only qualitative, but also quantitative agreement between the simulations and the measurements.

**Figure 8:**
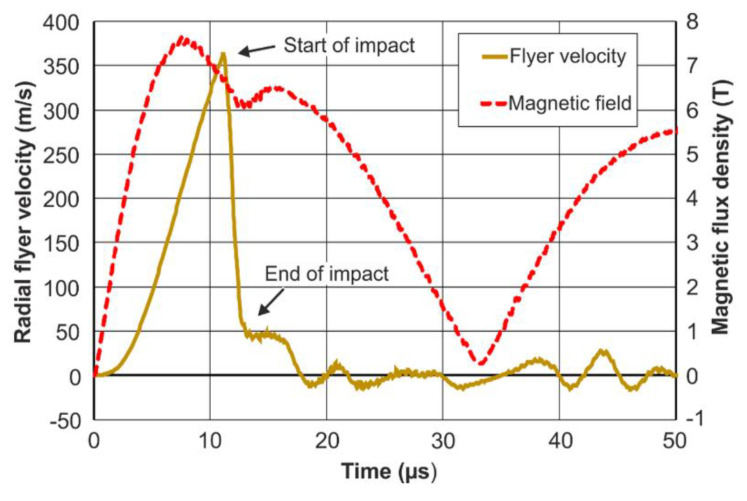
Radial velocity of the flyer edge and the magnetic field during the process. The magnetic field was evaluated at 0.1 mm from the field shaper edge and scaled with the mentioned factor of 0.3 for consideration of the borehole in the field shaper. The results are from the LS-DYNA simulation.

The numerical simulations allow for a detailed analysis of the interactions between the flyer deformation, the impact between the flyer and the parent, and the magnetic field dynamics. In Figure 8, the radial velocity of the flyer edge is plotted over time. It can be seen that the deformation of the flyer starts after with some delay and only after a certain level of magnetic field has been established. The induced stresses caused by the so-called magnetic pressure then exceed the initial flow stresses of the flyer material. The flyer is then rapidly accelerated until the standoff between the flyer and the parent is overcome. The contact of the inner flyer surface with the parent surface takes place at the maximum flyer velocity. It needs to be noted that, in the numerical simulations, the flyer velocity is determined for the flyer edge, which is obviously higher than at the PDV-measurement position a few millimeters along the flyer axis. This velocity peak is followed by a sudden deceleration, during which the flyer and the parent are slightly compressed, and a high contact pressure is formed. The deceleration phase takes only a few microseconds and its end can be identified by the local minimum of the magnetic field curve. This confirms the experimental results (Figure 4) and shows once again the potential of the magnetic field measurements for an in-depth analysis of MPW processes. The welding between the flyer and the parent was not modeled in the present LS-DYNA simulations and the flyer undergoes some vibrations after impact. This can be the reason for some of the deviations between simulations and experiment in the second and third half-wave of the current pulse shown in Figure 6. However, it is generally accepted that, among other parameters, the flyer’s radial impact velocity and the phase of impact between the joining partners have a paramount effect on the weld formation. Therefore, the correlation between the flyer deformation curve and the magnetic field measurements and the different characteristics for successful and unsuccessful welding trials are promising indications for the further development of this CMR-measurement method.

## 4. Conclusions and Outlook

This proposed method of measuring the magnetic field dynamics between the tool (coil or field shaper) and the movable (flyer) workpiece during electromagnetic forming by means of a CMR-B-scalar sensor gives valuable information about the phenomena which take place during electromagnetic tube forming or MPW processes. The experimental results and numerical simulations showed that the magnetic field waveform differs from the current pulse: The first half-wave pulse of the magnetic field has two peaks with a minimum. Its time-position depends on the charging energy of the capacitors and is associated with the collision of the flyer with the parent. Together with the first peak maximum and its time-position, this characteristic could be an indication of the welding quality. Such a nondestructive method of MPW process evaluation based on magnetic field dynamics measurement could also be used in combination with the peel testing method. Moreover, after performing a set of reference MPW experiments for different setups and materials, the magnetic field measurement system could be applied in future for the monitoring of MPW processes of known materials. Its advantage is that this measurement technique is fast and can be used inline. Moreover, the time of collision between the flyer and the parent and therefore the approximate collision velocity can now be determined without the use of elaborate measurement equipment like PDV or complex numerical simulations.

It was also concluded that the CMR-B-scalar probe with the measurement module has the advantage of measuring the magnetic field independently of its direction, which is contrary to well-known Hall, magnetoresistive, or loop sensors. This allows for an easier installation of the sensor into the measuring position, because it does not require an exact orientation of the sensor with respect to the magnetic field direction. Moreover, the CMR-B-scalar sensor is small and thus can be applied for measuring the magnetic field locally. In addition, the measurement module of the system has a sufficient time resolution to measure fast magnetic field changes.
